# 

**Published:** 1878-02

**Authors:** 


### Editorial.

### MISSISSIPPI VALLEY DENTAL ASSOCIATION.

The Thirty-Fourth Annual Meeting of the Mississippi Valley Dental Association, will be held in the Ohio Dental College at Cincinnati, commencing Wednesday, March 6th, 1878, at ten a. m., and continuing three days.

SUBJECTS FOR DISCUSSION.

First.—Dental hygiene.

Second.—Dental medication, local and general.

Third.—Affections accompaning first dentition.

Fourth.—Artificial dentures.

Fifth.—Operative dentistry.

Sixth.—Chemical processes that occur in the mouth, and its fluids.

Seventh.—Dental education and professional culture,

Eighth.—Suppuration of the gums and its treatment.

The executive committee would respectfully request a full attendance of the members.

A cordial invitation is extended to all the profession to attend these meetings.

### THE OHIO DENTAL COLLEGE ASSOCIATION.

The stockholders and members of the Ohio Dental College Association, will hold their annual meeting on Tuesday, March 5th, at two o'clock p. m. It is very desirable that all interested be present; as at every meeting of this body, there is business to transact, that involves not only the interest of the College, but the welfare of the profession.

Every member of this association is not only pecuniarily, responsible for the welfare and prosperity of the College, but for the cause of dental education in this section of the country.

The property of the College belongs to the stockholders and they should see to it that the Institution is well sustained and its management placed in the hands of those who will make it most efficient for good. The educational interests of the profession call for special attention just at this time.

*THE DENTAL REGISTER,*

A MONTHLY MAGAZINE DEVOTED TO THE INTERESTS OF

THE DENTAL PROFESSION.

**Terms Reduced to $2.50 per Year. Single Copies 25c.**

*EDITED BY PROF. J. TAFT, D. D. S.,*

AND PUBLISHED BY

SPENCER & CROCKER, Ohio Dental Depot.

The volume commences in January and closes in December.

The REGISTER being issued monthly is always fresh, therefore indispensable to the practitioner who desires to keep posted up with the new inventions and improvements which are daily developed. Neither expense nor effort will be spared to give value and variety in its contents. Articles will be illustrated with well executed engravings whenever they will add to the interest or assist to explanation.

Its extensive circulation and frequency of issue make it one of the BEST DENTAL ADVERTISING MEDIUMS in the United States.

All subscriptions must begin with January or July.

Local or special notices, five lines or less, will be inserted for $3.00 each insertion; over five lines, 50 cts. per line.

All advertising bills payable in advance, or at furthest, after the issue of the first number containing the advertisement. All transient advertisements to be paid for in advance.

☞CONTRIBUTIONS SOLICITED.

Exclusively Editorial Communications should be addressed to Editor.

The latest number of the REGISTER may be ordered with or without other goods at any time, and all letters should be addressed to

***SPENCER & CROCKER,***
Ohio Dental Depot, Cincinnati, O.

